# Effects and repercussions of local/hospital-based health technology assessment (HTA): a systematic review

**DOI:** 10.1186/2046-4053-3-129

**Published:** 2014-10-28

**Authors:** Marie-Pierre Gagnon, Marie Desmartis, Thomas Poder, William Witteman

**Affiliations:** 1 Centre de recherche du CHU de Québec, 10 rue de l’Espinay, D6-726 Quebec City, QC G1L 3L5, Canada; 2 Faculty of Nursing, Université Laval, Quebec City, QC, Canada; 3 UETMIS, Hôpital Hôtel-Dieu, CHUS, Sherbrooke, QC, Canada; 4 Centre de recherche du CHUS, Sherbrooke, QC, Canada

**Keywords:** Health technology assessment, Local/hospital-based HTA, Impact of HTA activities, Hospital budget, Perceptions of stakeholders, HTA units, Internal committee, Mini-HTA, Ambassador model

## Abstract

**Background:**

Health technology assessment (HTA) is increasingly performed at the local or hospital level where the costs, impacts, and benefits of health technologies can be directly assessed. Although local/hospital-based HTA has been implemented for more than two decades in some jurisdictions, little is known about its effects and impact on hospital budget, clinical practices, and patient outcomes. We conducted a mixed-methods systematic review that aimed to synthesize current evidence regarding the effects and impact of local/hospital-based HTA.

**Methods:**

We identified articles through PubMed and Embase and by citation tracking of included studies. We selected qualitative, quantitative, or mixed-methods studies with empirical data about the effects or impact of local/hospital-based HTA on decision-making, budget, or perceptions of stakeholders. We extracted the following information from included studies: country, methodological approach, and use of conceptual framework; local/hospital HTA approach and activities described; reported effects and impacts of local/hospital-based HTA; factors facilitating/hampering the use of hospital-based HTA recommendations; and perceptions of stakeholders concerning local/hospital HTA. Due to the great heterogeneity among studies, we conducted a narrative synthesis of their results.

**Results:**

A total of 18 studies met the inclusion criteria. We reported the results according to the four approaches for performing HTA proposed by the Hospital Based HTA Interest Sub-Group: ambassador model, mini-HTA, internal committee, and HTA unit. Results showed that each of these approaches for performing HTA corresponds to specific needs and structures and has its strengths and limitations. Overall, studies showed positive impacts related to local/hospital-based HTA on hospital decisions and budgets, as well as positive perceptions from managers and clinicians.

**Conclusions:**

Local/hospital-based HTA could influence decision-making on several aspects. It is difficult to evaluate the real impacts of local HTA at the different levels of health care given the relatively small number of evaluations with quantitative data and the lack of clear comparators. Further research is necessary to explore the conditions under which local/hospital-based HTA results and recommendations can impact hospital policies, clinical decisions, and quality of care and optimize the use of scarce resources.

## Background

While health technology assessment (HTA) is often done at a national or international level, many local health services and hospitals consider that it makes sense to move the assessment closer to the point of care, where the costs, impacts, and benefits of technologies can be directly assessed. This is justified by the fact that many decisions regarding health technologies (prioritization, investment, adoption, and disinvestment) are made at the local/hospital level [1]. This is also the result of an increasing awareness that specific organizational contexts should be taken into account when assessing health technologies [2].

With the emergence of HTA activities in hospitals, the Hospital Based HTA Interest Sub-Group was created within the HTAi—the international scientific and professional society for HTA—in 2006. In 2008, this sub-group elaborated a conceptual model to classify the different approaches for performing HTA within hospitals around the world [1]. Four different approaches were described: 1) ambassador model, 2) mini-HTA, 3) internal committee, and 4) HTA unit. The ambassador model seeks to promote changes in practice through a specific HTA dissemination approach. In this approach, interested clinicians who are recognized as opinion leaders play the role of ambassadors of the HTA message within health-care organizations at regional and local levels. Mini-HTA is a management and decision support tool that consists of questions about the technology, the patient, the organization, and the financial aspects [3]. The mini-HTA is usually done by a single professional who often participates in the assessment process, collecting data at the hospital level in order to inform decision makers. The internal committee consists in many cases of an *ad hoc* multidisciplinary group representing different perspectives, in charge of reviewing evidence and making recommendations to the health-care organization. The HTA unit represents the most structured model for hospital-based HTA. It is a formal organizational structure with specialized HTA personnel working on a full-time basis on the production of HTA material of high scientific quality.

According to a recent survey, the number of hospitals performing HTA is growing around the world [4]. While local/hospital-based HTA has been in place for a few decades in some jurisdictions, there is very limited knowledge of its effects on decisions regarding health technologies. Thus, it is important to review current evidence about local/hospital-based HTA in order to inform future initiatives.

This paper presents a systematic review of the effects and impact of local/hospital-based HTA reported in the literature on decision-making or management. The main questions that guided this review are the following: 1) Have HTA recommendations been accepted and implemented? 2) What expenses and savings are related to HTA activities and their recommendations? 3) What are the perceptions of various stakeholders towards local/hospital HTA? We also compiled information about strengths and weaknesses of different approaches of local/hospital-based HTA, as well as barriers and facilitators with respect to the implementation of their recommendations, as secondary outcomes.

## Methods

This review adheres to the Preferred Reporting Items for Systematic Reviews and Meta-Analyses (PRISMA) checklist [5] (see Additional file 1).

### Screening and selection

A review protocol was established, based on a previously published knowledge synthesis [6]. We searched for qualitative, quantitative, or mixed-methods studies with empirical data about the effects or impact of local/hospital-based HTA. We did not use date or language limits. Studies were excluded if they were not about local/hospital HTA activities; did not provide data concerning the impact of HTA activities on decision-making or management, budget, or perceptions of stakeholders as regards HTA activities; and were not based on an empirical analysis of the effects or impact of HTA activities or recommendations.

An information specialist (WW) developed the search strategy and performed literature search in two databases (PubMed and Embase; see Additional file 2). Other literature were identified through citation tracking of the included studies. Two members of the team (WW, MD) independently reviewed titles and abstracts for relevance, with the intervention of a third reviewer (MPG) in case of discrepancy. Teams of two reviewers (MD, TP, WW, and MPG) then independently conducted full-text reviews for eligibility; any disagreement was resolved by discussion among reviewers.

### Extraction of data

The same teams of two reviewers independently extracted the following information from the selected studies: general characteristics of the studies (country, methodological approach); local/hospital HTA approach and activities; use of conceptual framework or model; reported effects and impacts of local/hospital-based HTA; factors facilitating/hampering the use of hospital-based HTA recommendations; perceptions of stakeholders regarding local/hospital HTA activities (benefits and concerns); and resources needed for implementing hospital-based HTA. We also assessed study quality using the *Mixed Methods Appraisal Tool* (MMAT) [7]. Due to the great heterogeneity among studies, we conducted a narrative synthesis of their results [8].

## Results

A total of 707 potentially relevant articles were identified from the main search strategy, of which 15 met the eligibility criteria and were included. Other search strategies identified four additional eligible publications, but one of them was an update of an included study. Consequently, a total of 18 studies were included, described in 19 papers. The study selection process is presented in Additional file 3.

### Overview of studies

More than half of the studies were from the USA (*n* = 7) and Canada (*n* = 5). Other studies were from Denmark (*n* = 2), Austria (*n* = 2), and Australia and France (*n* = 1 each). Over half of the articles (10/18) were published since 2005 and a third (6/18) since 2010. The six articles published prior to 2000 were all from the USA, but one study was conducted in Canada [9].

#### Purpose and methods of the studies

Four studies surveyed hospitals in a country or in part of a country to explore how HTA was used in decision-making for the introduction of technological innovations. They used questionnaires [9-11] or semi-structured interviews [12]. Five studies used a case study to report the experience of use of HTA in a particular hospital [13-15] or in a few hospitals [16,17]. In all of these studies, with the exception of two [15,16], HTA was performed by committees of different types, often of an *ad hoc* nature.

Six studies reported an analysis of the outcomes of an HTA program or unit and the impact of their reports and recommendations. In Canada, Poulin et al. [18] conducted a retrospective analysis of the outcomes of an HTA program (over a 5-year period) and of the kinds of decisions that were made based on local committee recommendations. McGregor and Brophy [19,20] evaluated the impact of 55 HTA reports that their Technology Assessment Unit (TAU) produced during 8 years of service. They used interviews with local administrative and clinical decision makers as well as document analysis to evaluate the impact of HTA reports and recommendations on hospital policy decision-making and hospital spending. Other impact studies focused on an HTA unit or committee that covered many hospitals. For instance, Bodeau-Livinec et al. [21] studied the Committee for the Assessment and Dissemination of Technological Innovations (CEDIT in French), an HTA unit that covers a network of 39 university hospitals located in the Paris region. Similarly, the study by Lee et al. [22] illustrated the impact of the activities of an HTA unit in a health region in Alberta (Canada). Finally, Schumacher and Zechmeister [23,24] focused on the impact of an HTA research program that produces various types of reports for decision makers at different levels of the health-care system in Austria, including hospitals.

Using the survey approach, two studies assessed the use of mini-HTA in the introduction of new technologies in the Danish hospital sector [3] or in a major public hospital in Denmark [25]. The study by Rashiq et al. [26] evaluated the Alberta Ambassador Program put in place to inform clinicians about current research evidence on the management of chronic non-cancer pain, using a pre- and post-session questionnaire.

#### Quality of the studies

The assessment of the quality of studies can be found in Additional file 4. Even if the assessment of the quality of studies was conducted using the MMAT, we decided not to take this into account in the interpretation of our results due to the exploratory aim of the review. Overall, studies conducted after 2000 were of better quality than earlier studies.

### Reported effects and impacts of local/hospital-based HTA activities

Given the great heterogeneity among studies, notably in terms of approaches for performing HTA, we report their effects and impacts according to the four categories proposed by the Hospital Based HTA Interest Sub-Group [1] (ambassador model, mini-HTA, internal committee, and HTA unit). However, we found a wide range of “committees” in the studies reviewed. Their composition and structure varied across studies (from *ad hoc* committees to very structured ones). In this review, we distinguish committees from HTA units that are formal organizational structures with dedicated HTA personnel.

#### HTA committees

Reported effects and impacts of activities of different types of HTA committees on decision-making (or management) are presented in Table 1. As the only financial aspect reported in some of these studies [12,13] is the minimum cost related to a technology for deciding whether to assess them, we will not create a specific section here for financial impact.

**Table 1:** Reported effects and impact—HTA committees

**Reference and country**	**Methods/participants**	**Type of committee (structured or unstructured (*ad hoc*))**	**Type of impact**
Cram et al. 1997 USA [10]	Survey/33 clinical engineering departments throughout the USA	Not specified	**On decision-making (or management)**
		23/27 committees doing HTA are multidisciplinary	• Several respondents used HTA to cut costs and provide more standardization.
			• The HTA process was seen by some respondents as allowing broader input into decision-making processes.
			**On clinicians’ or other stakeholders’ perceptions**
			• 20/25 (80%) who used an HTA system felt it was a useful tool.
			• Main problems perceived in HTA processes: internal politics; lack of understanding that could lead committees to make poor decisions
Luce and Brown 1995 USA [12]	Interviews/48 participants from 30 organizations (hospitals, health maintenance organizations, and third-party payers)	Not specified	**On decision-making (or management)**
		For hospitals: multidisciplinary committees; formulary committees; department chiefs	• Hospital decision makers used HTA almost exclusively for making purchasing decisions and as a means of controlling expenditures.
			• Decisions were based on financial assessment with little or no formal evaluation of changes in patient outcomes or medical practice patterns.
			• Purchase or non-purchase recommendations were rarely contravened by management and were distributed to relevant departments throughout the organization.
			**Financial**
			• New technologies priced over a predetermined threshold (US $100,000 or $250,000) were all assessed prior to purchase.
Menon and Marshall 1990 Canada [9]	Survey/50 (59.5%) teaching hospitals across Canada	Structured: 23/50	**On decision-making (or management)**
			• 34/43 hospitals practicing HTA stated that information produced was used in making decisions about new technology acquisition.
			**On clinicians’ or other stakeholders’ perceptions**
			• 76% of respondents thought that a formal management structure for HTA should exist in teaching hospitals.
Patail and Aranha 1995 USA [13]	Case study/1 major teaching hospital	Structured	**On decision-making (or management)**
			• Of 16 technologies formally approved in 1988–1993, 13 were implemented.
			• HTA allowed engineers and decision makers not to take the information provided by manufacturers and vendors for granted.
			**Financial**
			• Technologies over $500,000 were assessed.
Poulin et al. 2012 Canada [18]	Case study of HTA program outcomes	Structured	**On decision-making (or management)**
			• Of the 68 technologies for which a HTA was requested, 15 were incomplete and dropped, 12 were approved, 3 were approved on an urgent/emergent basis, 21 were approved for “clinical audit” on a restricted basis, 14 were approved for research use only, and 3 were referred to additional review bodies.
			• Decisions based on local HTA program recommendations were rarely “yes” or “no”. Many technologies were given restricted approval, with full approval contingent on satisfying certain conditions such as clinical outcomes review, training protocol development, or funding.
			**Financial**
			• Cost was the first reason to reject a technology, followed by health gain.
Rosenstein et al. 2003 USA [11]	Survey/19 hospitals in western USA	Structured: 42%	**On decision-making (or management)**
		*Ad hoc*: 48%	• 28% of HTA committees had direct responsibility for approval.
			• While committees did not have final decision-making power, their recommendations were appropriate and well integrated with the hospital’s overall mission and strategic plan.
Saaid 2011 Australia [17]	Multicase study/4 hospitals (3 private for-profit, 1 public)	1/4 has a formal committee	**On decision-making (or management)**
		3/4 have a product review committee	• The impact of HTA as a support tool for decision makers was minimal.
			• Decisions in private for-profit hospitals were informal and driven by business strategy and cost-effectiveness of the technology.
			• For the public hospital, HTA was a requirement in decision-making, but the process was new.
			**On clinicians’ or other stakeholders’ perceptions**
			• Ignorance/unfamiliarity with HTA.
Weingart 1995 USA [14]	Case study/1 major teaching hospital	*Ad hoc*	**On decision-making (or management)**
			• The technology assessed was qualified as an engineering disaster for various reasons:
			• Decision makers did not go far enough in their discussions to evaluate the institutional strategy or strategic implications of the technology. They lacked expertise in assessing feasibility and profitability.
			• Members of the committee (only physicians) were too optimistic despite limited data.
			• The mandate of the committee was too narrow and did not include comparison with alternative technology.
			• The process was not structured enough (*ad hoc* structure), and there was no official strategic plan in place at the hospital.

##### Impact on hospital policies and management

A survey conducted in 30 organizations (including hospitals, health maintenance organizations (HMOs), and third-party payers) in the USA in 1995 [12] showed that decision makers used HTA to inform purchasing or coverage decisions regarding new and expensive technologies and drugs, as a means to better use resources. Decision makers usually followed the purchase or no purchase recommendation made by the assessment committee. However, with the exception of pharmacy committees, decisions were based on financial evaluation with little or no formal evaluation of changes in patient outcomes or medical practice patterns. A survey in teaching hospitals across Canada conducted in 1990 [9] found that HTA was common in teaching hospitals (43/50), although it took various forms. Thirty-four of the 43 hospitals practicing HTA stated that information from HTA was used in decision-making about new technology acquisition. However, only 23 hospitals had a formal management structure for HTA. In the Cram et al. study [10] of clinical engineering departments throughout the USA conducted in 1997, 80% of the respondents (20/25) who used an HTA process felt that it was a useful tool and several of them had used it to cut costs and provide more standardization. The benefits of different HTA processes varied from hospital to hospital, but some respondents stressed that the HTA process informed purchase requests to be evaluated by a multifunctional team, which allows for broadening the input involved in decision-making processes [10].

In a case study from 1995 concerning the impact of the assessment of biliary lithotripsy by a task force of physicians in a major teaching hospital in the USA, Weingart [14] reported that the works of this committee have led to purchasing of the technology, which was later described as an engineering disaster. The author outlined many reasons for this failure: the exaggerated optimism of physicians in the task force, despite limited data in published reports; a mandate that was too narrow and did not include comparisons with alternative technologies; the lack of expertise of decision makers in assessing feasibility and profitability; and an assessment process that was not structured enough.

Patail and Aranha [13] reported the impact of the work of a multidisciplinary assessment team, including managers and biomedical engineers, in a US hospital in 1995. Their results showed that of 16 technologies formally approved by the assessment team between 1988 and 1993, a total of 13 had been implemented. According to these authors, HTA made it possible for decision makers to avoid taking the information provided by manufacturers and vendors for granted.

In 2003, a survey among 19 hospitals in the western part of the USA [11] revealed that although 90% of hospitals reported the existence of a specific staff responsible for providing formal reviews, only 42% had a dedicated technology assessment committee. Among these committees, 28% had direct responsibility for technology approval. Even if committees did not make the final decision, respondents reported that their recommendations were well integrated into the hospital’s mission and strategic plan.

Recently, the multiple case study by Saaid [17] examined the use of HTA in decision-making processes for acquiring new health technologies in four selected hospitals (three not-for-profit private hospitals and one public hospital) of southeast Queensland (Australia). The results showed that the impact of HTA as a support tool for decision makers was relatively minimal. Decisions in private hospitals were informal and driven by business strategy and the cost-effectiveness of the technologies and also significantly influenced by physicians. In the public hospital, HTA was a requirement in decision-making, and a formal committee was in place, but it was at an early stage of development.

For their part, Poulin et al. [18] analyzed the outcomes of a local HTA program in Alberta (Canada) over a 5-year period. They reported that decisions based on recommendations of this program were rarely “yes” or “no” but offered many approval options between full acceptance and rejection. Many technologies have received restricted approval, with full approval contingent on satisfying conditions such as clinical outcomes review, training protocol development, or funding.

#### HTA unit

##### Impact on hospital policies and management

Reported effects and impact of activities of HTA units are presented in Table 2. Four studies of this category analyzed the impact of HTA reports and recommendations on hospital policies and implementation. The study by McGregor and Brophy [19,20] evaluated the impact of 55 reports issued by the TAU of the McGill University Health Center (MUHC) between 2004 and 2011. Of the 63 recommendations produced in these reports, 45 (71%) have been accepted and incorporated into hospital policy. The most frequent reason for recommendations not being accepted was failure to identify administrative responsibility to carry this out.

**Table 2:** Reported effects and impact—HTA units

**References and country**	**Methods**	**Type of impact**
Bodeau-Livinec et al. 2006 France [21]	Semi-directive interviews and survey	**On decision-making (or management)**
		• 10 of 13 recommendations had an impact on theintroduction of the technology in health organizations.
		• One recommendation appears not to have had an impact. The impact of two technologies was impossible to assess.
		**Financial**
		• The main criterion upon which to base a new technology introduction decision on HTA is the cost. Some medical specialties were more concerned by CEDIT’s work than others—cardiology and medical imaging, for instance.
		**On clinicians’ or other stakeholders’ perceptions**
		• Interviewees viewed the CEDIT as very scientifically reputable. HTA recommendations were used as decision-making tools by administrative staff and as negotiating instruments by physicians in dealing with management.
Lee et al. 2003 Canada [22]	Case study (review of document and structured consultation)	**Financial**
		• Example of one evaluation to address the issue of arthroplasty operations. Savings were estimated at CAN $1 million annually through orthopedic supply standardization and a new contract with vendors.
		**On clinicians’ or other stakeholders’ perceptions**
		• High level of interest for a locally focused HTA and implementation unit.
McGregor 2012 Canada [19]	Impact study using mixed methods (interviews and financial analysis)	**On decision-making (or management)**
		• Impact of 55 HTA reports produced (2004–2011): Of 63 recommendations, 45 (71%) have been accepted and incorporated into hospital policy.
Update of McGregor and Brophy 2005 Canada [20]		• Most frequent reasons for recommendations not being accepted: failure to identify administrative responsibility to carry this out, lack of funds, complex administrative changes, technology already implanted, technology which would potentially render the hospital vulnerable to legal action.
		**Financial**
		• 19 accepted reports have resulted in conservation of hospital resources.
		• The extent of these savings could be estimated in the case of 15 reports: estimated overall savings of CAN$ 9,840,270.
		• Over the 8 years of full functioning of the HTA unit: average annual quantifiable savings have been CAN$ 1,140,958.
Mitchell 2010 USA [15]	Case studies	**On decision-making (or management)**
		• Two examples of local data integrated into hospital-based HTA. In both case studies, important differences were found among the hospitals. These differences affected the prioritization of different attributes of a technology and could result in different conclusions being drawn about how the technology should be used at each hospital, even within the same health-care network.
Veluchamy and Alder 1989 USA [16]	Case study	**On decision-making (or management)**
		• The HTA units helped decision makers integrate patient needs and medical staff interests and capabilities with the hospital’s resources (i.e., staff, facilities, financing).
		• It speeded up the delivery of newly developed treatment technologies (9–12 months as compared to 24–36 months before HTA implementation). It identified the most promising technologies and coordinated their acquisition and implementation.
		• It provided better access to these technologies for patients and reduced length of stay (42% reduction for laser angioplasty).
		**On clinicians’ or other stakeholders’ perceptions**
		• Physicians derived personal and professional satisfaction from participation in the HTA units. These units have improved relations between medical staff and hospital management (better communication and physicians’ needs better fulfilled).
Schumacher and Zechmeister 2013 Austria [23]	Impact study using mixed methods (interviews, questionnaire, download analysis, etc.)	**On decision-making (or management)**
		• Hospital associations used HTA for investment/reimbursement decisions, treatment guidelines, and budget allocation, as well as for the preparation of negotiations.
		• Various pressure groups, such as the pharmaceutical industry and the professionals’ association, could explain the inability to implement some HTA recommendations.
		• With the exception of the rapid technology assessment program for single hospital procedures, selective use of HTA reports was identified, rather than standardized inclusion of HTA into the processes.
		**Financial**
		• Several technologies, identified as showing patterns of over-usage, were used more restrictively after the HTA report was published, leading to a decrease in expenditure. Expenditure decrease accounted for at least several million euros for single hospital associations.
		**On clinicians’ or other stakeholders’ perceptions**
		• Clearest evidence was available for the “awareness” impact category, while references regarding “acceptance” were rarely mentioned. The LBI-HTA was usually seen as a vehicle for simple cost containment and rationing, rather than a tool supporting redistribution of resources into evidence-based technologies.
Zechmeister and Schumacher 2012 Austria [24]	Impact study using mixed methods (administrative data analysis and interviews)	**On decision-making (or management)**
		• 5 full HTA reports and 56 rapid technology assessments were used for reimbursement decisions, while 4 full HTA reports and 2 rapid assessments were used for disinvestment decisions and resulted in reduced volumes and expenditure. There were 2 full HTA reports showing no impact on decision-making. Impact was most evident for hospital technologies.
		• In 48% of reports produced for reimbursement/investment decisions, the recommendation and decision were totally consistent. In 40% of reports, technologies that were not recommended were included on certain conditions, while the decision was more restrictive than the recommendation for 12% of reports.
		**Financial**
		• Several millions of euros were saved due to HTA recommendations. For disinvestment decisions, cost savings were about 3 million euros per report, with huge variation (0–12 million). Savings were frequently for more than one hospital (regional hospital associations).

In France, Bodeau-Livinec et al. [21] assessed the perceptions of various stakeholders regarding the use of HTA recommendations produced by the CEDIT. Decision makers found these recommendations very useful and reported a good match between the recommendations and their implementation. Of the 13 recommendations produced, ten had an impact on the introduction of the technology in health organizations and only one did not have an impact; the impact of the two remaining recommendations was impossible to assess.

In two studies [23,24], Schumacher and Zechmeister analyzed the impact of the HTA research program of the Institute of Technology Assessment (ITA) and the Ludwig Boltzmann Institute for HTA (LBI-HTA) on the Austrian health-care system. In one study, they used a multidimensional framework based on seven impact categories: awareness, acceptance, policy process, policy decision, practice (clinical, reimbursement), final outcomes and economic impact, and, lastly, enlightenment [23]. Their results showed evidence of impact for all of the predefined categories, but particularly on hospitals where HTA is used for investment/reimbursement decisions, treatment guidelines, budget allocation, and the preparation of negotiation. For example, authors reported that the recommendation and decision were totally consistent for 48% of the reports produced for reimbursement/investment decisions. For 40% of the reports, technologies that had not been recommended were included based on certain conditions, while in 12% of the reports, the decision was more restrictive than the recommendation [24].

Veluchamy and Alder [16] described the many positive effects of an HTA unit in two hospitals of the Mount Carmel Health Region (USA). In fact, their case study reported integration of patient needs and medical staff interests and capabilities with the hospital’s resources, higher speed of delivery of newly developed treatment, better access for patients to these technologies, and reduced length of stay as consequences of the presence of the HTA unit. Finally, the study by Mitchell et al. [15] described two examples of how local data integrated into hospital-based HTA were used at the institutional level to inform decisions. In the first example, qualitative local data (staffing patterns and local preferences) had considerable bearing on technology choice (the selection of a new cardiac catheterization lab). In the second example, local outcomes data from administrative records were decisive in the decision on whether or not to continue telemedicine services in critical care units. In both case studies, important differences were found among the hospitals.

##### Financial impact

Among studies that reported financial impact of HTA activities, McGregor’s study [19] demonstrated that 19 accepted reports have resulted in conservation of hospital resources. To measure this impact, they considered that without a negative recommendation their hospital would have authorized the technology; hence, the recommendation translates into cost saving for the hospital. The extent of savings could only be estimated for 15 reports, which accounted for overall estimated savings of CAN$ 9,840,270. Over the course of 8 years in this HTA unit, the average annual quantifiable saving was CAN$ 1,140,958.

The financial impact of the Calgary Health Technology Implementation Unit was illustrated through an example of an evaluation that addressed the issue of arthroplasty operations in a health region of Alberta (Canada) [22]. The authors estimated that savings reached CAN$ 1 million annually through orthopedic supply standardization and a new contract with vendors.

The Schumacher and Zechmeister studies [23,24] showed that using HTA in decision-making resulted in various economic outcomes. First, several hospital technologies that had been assessed as showing patterns of over-usage were used more restrictively after the HTA report had been published, leading to a decrease in expenditure [23]. Interviews and analysis of administrative data showed that the impact of HTA recommendations translated into a global cost saving of several million euros for single hospital associations [24].

#### Mini-HTA

##### Impact on hospital policies and management

Although mini-HTA is widely used in hospitals in Denmark as the principal basis for decision-making, the Ehlers et al. study [3] reported that no decision makers based their decisions exclusively on them. Mini-HTA could ease technology implementation to a considerable degree; through their local participation in the analysis, stakeholders may acquire a better understanding of the new technology and become more willing to implement it [3]. The study by Folkersen and Pedersen [25] showed similar positive effects of the use of mini-HTA in one major Danish hospital: a greater level of contact between doctors and administrative staff and improved relationships between health professionals and economists, which have often been problematic due to the perception of competing priorities (quality vs. budget). A satisfaction rate of 77% with the HTA method among respondents has also been found in this study (Table 3).

**Table 3:** Reported effects and impact—mini-HTA and ambassador model

**References and country**	**Methods/participants**	**Mini-HTA or ambassador**	**Type of impact**
Ehlers and Jensen 2006 Denmark [3]	Survey/140 Danish hospitals	Mini-HTA	**On decision-making (management)**
			• Mini-HTA is used as a decision support tool at all decision-making levels within the Danish hospital sector.
			• No decision makers based their decisions exclusively on mini-HTAs (but always used them as a supplement).
			• In hospital management sectors, the mini-HTA was often the principal basis for decision-making.
			• A majority of decision makers stated that the mini-HTA eased implementation to a considerable or fair degree.
			**On clinicians’ or other stakeholders’ perceptions**
			• Advantages of using mini-HTAs:
			○ Based on HTA principles
			○ The form of the tool, be it a tabular form or a checklist
			○ The way the form or checklist was being used (flexibility, openness, and timing)
			• Disadvantages mentioned typically centered on insufficiency of the evaluation of the evidence base and the lack of quality control.
Folkersen and Pedersen 2006 Denmark [25]	Survey/1 of Denmark’s main public hospital	Mini-HTA	**On decision-making (management)**
			• The HTA method has improved the relationships between health professionals and economists, which were previously problematic due to the perception of different or opposing priorities (quality vs. budget). Both parties have become more understanding towards the roles and tasks of the other party due to the implementation of mini-HTA.
			**On clinicians’ or other stakeholders’ perceptions**
			• Overall, 77% of respondents were satisfied with the HTA method.
			• Some dissatisfaction concerning the HTA method included:
			○ Causing a too great and troublesome administrative burden
			○ Placing too much or exclusive emphasis on financial factors, while neglecting professional and technical aspects
			○ Causing limits on budgets, which in turn prevented the purchase of new equipment
			○ Financial questions were too difficult to answer for some hospital staff
Rashiq et al 2006 Canada [26]	Pre- and post-session questionnaires/pre: 130 participants; post: 79 (60.8%)	Ambassador model	**On decision-making (management)**
			• The ambassador program was successful in increasing awareness of the best evidence in chronic non-cancer pain management and positively influenced treatment decisions.
			• Some participants (35%) reported practice changes as a result of the workshops.
			• 70% indicated that an action plan has been developed following the workshop.
			• 80% indicated that they disseminated the material to other practitioners.
			**On clinicians’ or other stakeholders’ perceptions**
			• 99% indicated that the workshops had been a useful way of linking research to practice.

#### Ambassador model

The Rashiq et al. study [26] evaluated the Alberta Ambassador Program, which was seeking to promote changes in practice in rural areas and among isolated practitioners in Alberta, on the topic of chronic non-cancer pain (CNCP) management. The results of a pre- and post-evaluation of 2-h interactive sessions provided by the HTA ambassador showed that it was successful in increasing awareness of the best evidence in CNCP management and positively influenced treatment decisions. The evaluation showed that 35% of participants reported practice changes as a consequence of the workshops, 70% indicated that an action plan has been developed as a result of the program, and 80% indicated that they shared the material with other practitioners [26].

### Barriers and facilitators with respect to the success of local/hospital-based HTA and the uptake of recommendations

#### HTA committee

In a study of proto-HTA—that is the application of an HTA approach without using the term HTA—in the USA, internal politics was one of the most important hindrances in successfully applying the HTA process [10]. The large amount of time required to perform HTA was also seen as a problem by some respondents [10]. Furthermore, lack of understanding also led committees to make poor decisions on some occasions [10]. Another study from the USA [12] reported that hospitals used technology assessment to control expenditures, and there was little or no evaluation of patient outcomes or medical practice patterns. This study also mentioned the importance of producing a credible and useful assessment. Patail and Aranha [13], Luce and Brown [12], Menon and Marshall [9], and Weingart [14] underscored the importance of a multidisciplinary team with strong corporate leadership and the use of a structured process.

According to a study by Rosenstein et al. [11], organizations that demonstrated success in evaluating technology had the following two characteristics: a multidisciplinary composition of the committee that included physician representation and an organizational commitment to dedicating resources to support the technology assessment program. Technology assessment should also be an important component of the hospital’s strategic plan. According to Poulin et al. [18], the use of multicriteria decision tools and patient/public input were also important factors facilitating the use of recommendations. Saaid [17] suggested that the HTA committee or unit needed to be independent to prevent too close a connection between HTA actors and decision makers.

#### HTA unit

According to Mitchell et al. [15], clinicians should be consulted before decision-making criteria are finalized because they have crucial insight into how a device or technology can increase or decrease the efficiency of care, which ensures better “buy-in” of the finished product [15]. They also mentioned the importance of documenting costs and benefits and considering the attributes that are important for doctors and managers. Among factors explaining uptake of the recommendations produced by their HTA unit, McGregor [19] mentioned the role of a policy committee that includes representatives of nurses, physicians, allied health professionals, patients, and administrators and that reflects the values of institutional members. McGregor and Brophy [19,20] also mentioned transparency, relevance of topics for hospital management, fairness, and timeliness of reports (ideally delivered within 6 months) as factors increasing the uptake of HTA recommendations. Clear identification of the authorities responsible for the initiation of the report and for its acceptance, as well as the individuals in charge of carrying out its recommendations, was also recommended [19]. Lastly, Lee et al. [22] underscored local focus, involvement in implementation of HTA recommendations, and collaboration with academia as factors related to the success of the Calgary Health Technology Implementation Unit in Alberta.

Among the barriers to the uptake of HTA recommendations, Bodeau-Livinec et al. [21] mentioned time taken to complete investigative procedures, poor knowledge of recommendations, and recommendations becoming obsolete as a result of developments in knowledge and technology. These authors also stressed that some respondents felt that the HTA unit (CEDIT) was too closely connected with decision-making departments [21]. For their part, Schumacher and Zechmeister [23] reported the lack of acceptance of HTA based on stakeholders’ perception that it was more a vehicle for cost containment and rationing rather than a tool supporting redistribution of resources based on scientific evidence for a more efficient use of resources.

#### Mini-HTA

According to Ehlers et al. [3], mini-HTA facilitated implementation of recommendations because key stakeholders’ participation in the assessment favored a higher degree of ownership and willingness to implement the new technologies. Advantages of using mini-HTAs included the form of the tool, be it a tabular form or a checklist, and the way which it could be used (flexibility, openness, and timing). Disadvantages of mini-HTA typically centered on insufficient evaluation of the evidence base and the lack of quality control, too much emphasis on financial factors, and difficulty answering financial questions for some hospital staff [3].

#### Ambassador model

The Rashiq et al. study [26] demonstrated that developing a teaching strategy by a multidisciplinary team is a condition for the success of the ambassador approach addressed to rural health practitioners and administrators. The perceived credibility of the ambassadors as content and methodology experts has been shown to play a major role in the uptake of HTA recommendations. To provide one-page summaries of HTA evidence and to deliver locally interactive sessions are other factors facilitating the success of the ambassador approach as a way to transfer HTA knowledge. Good communication skills are also a factor of success [26].

## Discussion

Although HTA has been conducted at the local and hospital level for more than two decades, evidence from the scientific literature is very limited regarding its effects on decision-making as well as its impact on costs. This is mainly due to the fact that few evaluations have been conducted by those who are involved in local/hospital-based HTA (internal evaluation) and even fewer by people outside these organizations (external evaluation).

Nevertheless, most studies reviewed reported a positive impact of local/hospital-based HTA on decisions regarding the acquisition or withdrawal of health technologies in hospitals, as well as positive perceptions from managers and clinicians [3,9-11,13,16,18-25].

Each of the four HTA approaches for performing HTA (ambassador model, mini-HTA, internal committee, and HTA unit) corresponds to specific needs and structures and has its strengths and limitations. The literature shows that the ambassador model can impact clinicians’ decisions [27], but it remains a strategy that relies upon individual clinicians whose influence, interest, and availability may vary. However, this model is associated with minimal costs, essentially limited to support for training and networking of the ambassadors.

The structure (form or checklist) of mini-HTAs and their features (flexibility, openness, and timing) are greatly appreciated by decision makers [3]. However, insufficient evaluations and lack of quality control could be important disadvantages. Consequently, there may be some concern about transparency and partiality [3]. The costs of performing mini-HTAs in Denmark and elsewhere have not been documented in the scientific literature.

With respect to internal HTA committees, some authors expressed concerns about the fact that these committees may not have the expertise to appraise or synthesize scientific evidence adequately [10,14]. Moreover, risk for conflicts of interests may exist when evaluations are performed at the level of a clinical department rather than at the hospital level [28]. In this case, evaluations may be too narrow in scope and biased towards interventions performed by that department. Although the composition of internal committees varies from one hospital to another, members of such committees are already employed by the health-care organization, which limits the operating costs of these committees*.* According to several authors [9-14,18-20], the most efficient structure for an HTA committee would be a single, multidisciplinary committee that includes physician and nursing representation, members of the administration and finance sectors, and, eventually, patient representatives [18,20].

Lastly, the formal HTA unit, which is the most complex organizational structure for hospital HTA, presents several advantages, such as depth, high quality, and scientific rigor of the HTA process [6,15,16]. The fact that the HTA unit works in partnership with all stakeholders interested in the technology, and its relative independence from clinicians or hospital management, is also highlighted as a benefit of this kind of structure [20]. Nevertheless, its main disadvantage is the fact that the HTA unit requires investments in terms of salary and space for professionals, which poses a trade-off for hospital managers. Time is also needed to implement an HTA unit in a hospital because of the learning curve, but having an experienced HTA professional leading the unit can shorten this.

Assessing the impact of local/hospital-based HTA recommendations on decision-making can be challenging, particularly for technologies whose value is only perceived after several years of utilization [21]. In such cases, it is difficult to predict with certainty whether the dissemination of the technology would have been the same if the HTA were not carried out. Moreover, the presence of some incentives or circumstances that promote the dissemination of the technology may impede assessment of the impact of some HTA recommendations [21]. Other sources of information such as scientific publications may have an impact on the introduction of new technologies, making identification of the specific impact of HTA recommendations more difficult [29].

Although the first studies on local/hospital HTA were published almost 25 years ago, most of these experiences are recent. The limited evidence currently available on local/hospital HTA makes it difficult to evaluate its effects and impacts at the different levels of health service delivery. Besides, many of these evaluations were conducted internally, by people who are involved in HTA, introducing a potential bias. Further research is necessary to explore the conditions under which local/hospital-based HTA results and recommendations can impact hospital policies, clinical decisions, and quality of patient care and optimize the use of scarce resources. It would be necessary to conduct more independent studies that use high-quality qualitative and quantitative methods for evaluating the impact of HTA in several dimensions. One of the dimensions deserving special attention is the impact of HTA on patients, as they should benefit from optimal resource allocation based on scientific evidence.

Another shortcoming of the studies reviewed is the absence of a theoretical model to evaluate the impact of local/hospital HTA. The only exception is the study by Shumacher and Zeichmester [23] that uses a multidimensional conceptual model with seven impact categories. A framework for evaluating local/hospital HTA should consider its impact at different levels, starting with the uptake of HTA recommendations by decision makers and its effect on funding decisions, its effect on health-care professional practices, and ultimately its impact on health-care outcomes both short and long term. Such evaluation would however require significant resources due to the need to control many confounding factors.

### Study limitations

Although it provides a comprehensive synthesis of the effects and impact of local/hospital-based HTA conducted internationally, this review has some limitations. First, given that only published studies have been included in the review, some valuable studies concerning the impact of hospital-based HTA may have been overlooked. For example, we found an abstract about the potential impact of hospital-based HTA in Italy [30], but we were unable to obtain the full study report from the authors. Furthermore, many relevant abstracts are presented each year at the HTAi annual conference, such as the experience of the Hospital Clinic in Catalonia [31]. However, information reported in an abstract is often limited, making it difficult to include this information in our synthesis. However, we have consulted two other reviews that were published as reports and include gray literature such as conference abstracts and research reports [6,32]. Although those reviews present a few international experiences of local/hospital HTA that are not reported in our review, they do not provide new evidence on the specific topic of our review.

Second, the use of the model proposed by the Hospital Based HTA Interest Sub-Group [1] to classify the different approaches to conducting hospital-based HTA has limitations because it includes distinct features: organizational structure (HTA committee and HTA unit), HTA tool (mini-HTA), and dissemination strategy (ambassador model). Also, the structure and functions of HTA committees varied greatly between studies, and the early HTA experiences reported might not conform to the actual standards in this field. This leads to another limitation of this review due to the great heterogeneity between included studies. In fact, this is not possible to make comparisons between the different local/hospital HTA experiences because they represent distinct interventions. Further work is needed in order to refine the conceptualization of local/hospital-based HTA and provide a common understanding of what it is and how it could be evaluated. A third limitation to this review is the fact that we have not considered study quality in the interpretation of the results. Given that studies published after 2000 generally have higher quality, an update of this review should focus on the most recent literature on local/hospital HTA.

## Conclusions

This systematic review provides a basis for understanding how local/hospital-based HTA could impact decision-making regarding the introduction of new technologies in the health-care system. However, our capacity to evaluate the real impacts of local/hospital-based HTA is limited given the relatively small number of evaluations with quantitative data, the lack of clear comparators, and the fact that most evaluations are conducted internally. Further research, using rigorous methods and preferably conducted by external assessors, is necessary to understand the conditions under which local/hospital-based HTA results and recommendations can impact hospital policies, clinical decisions, and quality of care and optimize the use of scarce resources.

## Abbreviations

HTA: Health technology assessment; HTAi: Health Technology Assessment International; MMAT: Mixed Methods Appraisal Tool; TAU: Technology Assessment Unit; CEDIT (French): Committee for the Assessment and Dissemination of Technological Innovations; MUHC: McGill University Health Center; CNCP: Chronic non-cancer pain.

## Competing interests

The authors declare that they have no competing interests.

## Authors’ contributions

MPG and MD designed the review protocol, conducted full-text reviews for eligibility, extracted data from selected studies, conducted quality assessments, and drafted the manuscript. TP reviewed studies for eligibility, extracted data from selected studies, conducted quality assessments, and revised the manuscript critically. WW carried out the literature searches and reviewed studies for eligibility. All authors commented upon and approved the final version of the manuscript.

## Supplementary Material

Additional file 1 **PRISMA checklist.** Description of the items included in the reporting of the systematic review. Click here for file

Additional file 2 **Electronic search strategy.** Description of the search strategy used in PubMed and Embase. Click here for file

Additional file 3 **PRISMA flow diagram.** Diagram presenting the study selection process. Click here for file

Additional file 4 **Assessment of the quality of the studies.** Table presenting the study quality assessment score. Click here for file
