# Supplementary material for: Effects and repercussions of local/hospital-based health technology assessment (HTA): a systematic review
Source: Syst Rev. 2014 Oct 28;3:129. doi: 10.1186/2046-4053-3-129 (PMC4218945; doi:10.1186/2046-4053-3-129)
Supplement: Additional file 2 — Electronic search strategy. Description of the search strategy used in PubMed and Embase. [file 2046-4053-3-129-S2.docx]

**Additional file 2- Electronic search strategy**

PubMed Search Strategy:

((((Technology Assessment, Biomedical[MH] OR "Technology Assessment"[TIAB] OR HTA[TIAB] OR "Technology Assessment"[AD] OR HTA[AD]))) AND ((DECISION MAKING OR Decision Making, Organizational OR "Decision Support Techniques"[Mesh] or "decision aid*"[tiab] or "decision support"[tiab] or "health system"[tiab]))) AND ((administration, hospital[mh] or Health Facility Administration[mh] or hospital[tiab] or region*[tiab] or local*[tiab]))

EMBASE Search Strategy:

'technology assessment, biomedical'/exp OR 'technology assessment':ab,ti OR hta:ab,ti AND ('decision making':ab,ti OR 'decision making, organizational'/exp OR 'decision support techniques'/exp OR decision NEXT/2 aid* OR 'decision support':ab,ti OR 'health system':ab,ti) AND ('administration, hospital'/exp OR 'health facility administration'/exp OR hospital:ab,ti OR region*:ab,ti OR local*:ab,ti)
