# Supplementary material for: Effects and repercussions of local/hospital-based health technology assessment (HTA): a systematic review
Source: Syst Rev. 2014 Oct 28;3:129. doi: 10.1186/2046-4053-3-129 (PMC4218945; doi:10.1186/2046-4053-3-129)
Supplement: Additional file 3 — PRISMA flow diagram. Diagram presenting the study selection process. [file 2046-4053-3-129-S3.pdf]

PRISMA Flow Diagram

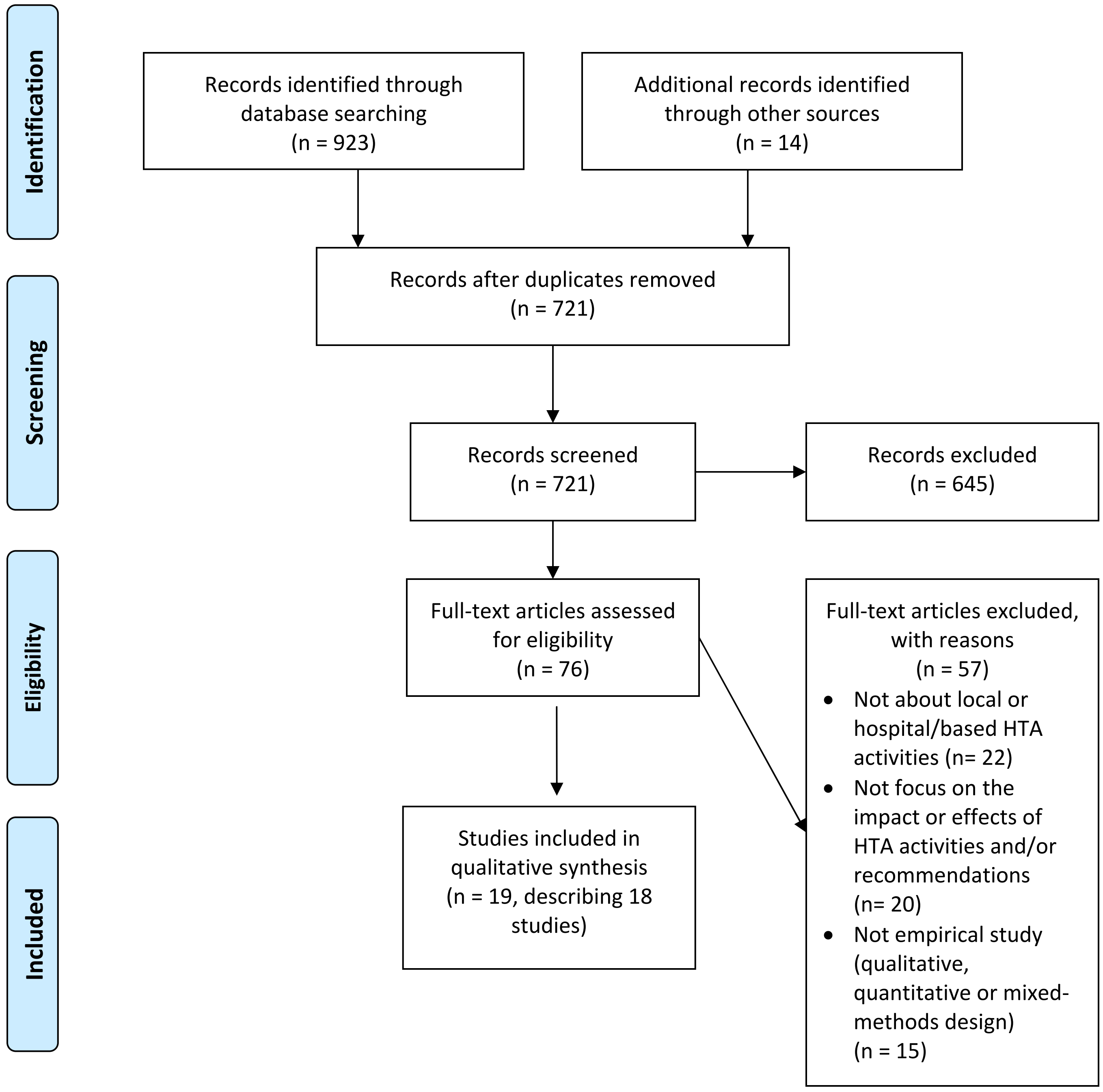

From: Moher D, Liberati A, Tetzlaff J, Altman DG, The PRISMA Group (2009). *P*referred *R*eporting *I*tems for *S*ystematic Reviews and *M*eta-*A*nalyses: The PRISMA Statement. PLoS Med 6(6): e1000097. doi:10.1371/journal.pmed1000097

For more information, visit www.prisma-statement.org.
