# Supplementary material for: Effects and repercussions of local/hospital-based health technology assessment (HTA): a systematic review
Source: Syst Rev. 2014 Oct 28;3:129. doi: 10.1186/2046-4053-3-129 (PMC4218945; doi:10.1186/2046-4053-3-129)
Supplement: Additional file 4 — Assessment of the quality of the studies. Table presenting the study quality assessment score. [file 2046-4053-3-129-S4.docx]

| **References** | **Country** | **Methodological approach** | | | **Appraisal of quality (Y : yes; N: no; U : Unclear NA: Not applicable)** | | | | | | | | | | |
| --- | --- | --- | --- | --- | --- | --- | --- | --- | --- | --- | --- | --- | --- | --- | --- |
|  |  |  | | | **Qualitative** | | | | **Quantitative descriptive** | | | | **Mixed Methods** | | |
|  |  | **Qualitative** | **Quantitative** | **Mixed** | **Sources of data relevant to address the research question** | **Process for analysing data relevant** | **Appropria-te considera-tion of the context or setting** | **Appropriate consideration to the researchers' influence through their interactions with participants** | **Sampling strategy relevant** | **Sample representative of the population under study** | **Appropri-ate measure-ments** | **Accepta-ble response rate (60% or above)** | **Design relevant to address the questions or objectives** | **Integration of qualitative and quantitative data relevant** | **Appropriate consideration given to the limitations associated with this integration** |
| Bodeau-Livinec et al. 2006 | France | Semi-directive interviews + case study |  |  | Y | Y | Y | N |  |  |  |  |  |  |  |
| Cram et al. 1997 | USA |  |  | Questionnaire including open ended questions | Y | Y | Y | N | N | N | N | N | N | N | N |
| Ehlers & Jensen 2006 | Denmark |  |  | Questionnaires including open ended questions | Y | N | Y | N | Y | Y | Y | Y | Y | Y | N |
| Folkersen & Pedersen 2006 | Denmark |  |  | Questionnaires including open ended questions | N | Y | Y | N | N | Y | Y | Y | Y | Y | Y |
| Lee et al. 2003 | Canada (Alberta) | Case study: review of documents, consultation among key individuals |  |  | Y | U | Y | N |  |  |  |  |  |  |  |
| Luce & Brown 1995 | USA | Case study Interviews |  |  | Y | U | U | N |  |  |  |  |  |  |  |
| McGregor 2012 (update of MacGregor & Brophy 2005) | Canada (Quebec) |  |  | Interviews, financial analysis | Y | U | Y | N | Y | Y | Y | Y | Y | Y | N |
| Menon & Marshall 1990 | Canada |  | Survey |  |  |  |  |  | Y | U | U | Y |  |  |  |
| Mitchell 2010 | USA | Case studies |  |  | U | U | Y | N |  |  |  |  |  |  |  |
| Patail & Aranha 1995 | USA | Case study |  |  | U | U | Y | N |  |  |  |  |  |  |  |
| Poulin et al. 2012 | Canada (Alberta) |  |  | Retrospective analysis of a HTA program over a 5-year period | Y | Y | Y | N | Y | Y | Y | N/A | Y | Y | N |
| Rashiq et al. 2006 | Canada (Alberta) |  |  | Pre- and post session evaluations: questionnaires including open-ended items | Y | Y | Y | N | Y | Y | U | Y | Y | N | N |
| Rosenstein et al. 2003 | USA (western part) |  |  | Questionnaires with closed and open-ended questions | Y | U | N | N | U | U | U | Y | Y | Y | N |
| Saaid et al 2011 | Australia | Multi case study |  |  | U | Y | Y | N |  |  |  |  |  |  |  |
| Schumacher & Zechmeister 2013 | Austria |  |  | Interviews, questionnaire, download analysis | Y | Y | N | N | U | U | U | Y | Y | Y | Y |
| Veluchamy & Alder 1989 | USA | Case study |  |  | U | N | Y | N |  |  |  |  |  |  |  |
| Weingart 1995 | USA | Case study |  |  | U | U | Y | U |  |  |  |  |  |  |  |
| Zechmeister & Schumacher 2012 | Austria |  |  | Administrative data from hospitals and health insurance funds, interviews | U | Y | Y | N | Y | Y | U | U | Y | Y | Y |
